# Notes

**Published:** 1898-02

**Authors:** 


### Notes.

By a singular coincidence armsol is recommended for hemorrhoids.

Dr. R. Harvey Reed has retired from the editorship of the *Columbus Medical Journal.*

The *Alabama Medical and Surgical Age* has moved from Anniston to Birmingham.

It is reported that Dr. George H. Noble will shortly erect here a sanitarium for the treatment of diseases of women.

An esteemed contemporary asks: "How to elevate the profession in the country?" What's the matter with dynamite?

Dr. George H. Stubbs, formerly of Atlanta, has located in Birmingham, Alabama, for the exclusive practice of the eye, ear, nose and throat diseases.

Dr. Don B. Bosworth of Atlanta, died in December, aged twenty-eight years. He was a young man of great promise, and his death is to be regretted.

The Tennessee Medical College, Knoxville, Tenn., was burned on December 3d. The building was comparatively new and cost $25,000; contents, $12,000.

Dr. Louis H. Jones and Dr. C. F. Benson have been appointed members of the Board of Health, to succeed Dr. F. W. McRae and Dr. H. P. Cooper, who have resigned.

Dr. Dunbar Roy has purchased a part interest in the Atlanta *Medical and Surgical Journal*, and will be hereafter identified with the editorial department of that excellent journal.

Dr. H. C. Wood, Professor of Therapeutics in the University of Pennsylvania, has assumed editorial charge of the *American Medico-Surgical Bulletin.* Dr. R. G. Eggles will remain as managing editor.

The American Association of Laryngologists, Otologists and Ophthalmologists, will convene at Atlanta, March 28. Dr. A. W. Calhoun is chairman of the committee of arrangements, and Dr. Dunbar Roy, secretary.

---

The profession has recently sustained the loss of the following well known members: Joseph O'Dwyer, the designer of the tubes used in intubation of the larynx; Ernest Hart, editor of the *British Medical Journal;* Tarnier, inventor of the axis-traction forceps; J. Berrien Lindsey.

---

The statement has been made, and is probably near the mark, that fifty per cent. of the people will shirk paying their doctor and will lower themselves to almost any mean subterfuge in order to save a few dollars. The belief would appear to be widespread, that physicians earn their fees easily, and they are in consequence looked upon as a fair game by that class of the community which likes to get something for nothing. A custom prevails in this country that ministers should be considered as free from any pecuniary obligation to the doctor for services rendered. This custom has been in existence for so long a time that the fact seems to have been forgotten that this free service is only an act of courtesy on the part of the physician, and not, as the minister imagines, by any means binding. The explanation for this state of affairs is not easy to give.—*Md. Med. Journal.*

---

A sample copy of the *Afro-American Beacon-Light* has been sent us with the request to give it notice in our columns. This we cheerfully do, and take occasion to felicitate the astute editor upon his admirable forethought in adapting his subject-matter to the needs of his patrons. That he is conversant with the characteristics of his subscribers, who can doubt after having seen the display ad. "next to pure reading matter," of the "Dixie Razor?" A gentleman's razor, American manufacture, 11–16 inches wide, hollow ground, hollow point (not only hollow itself but guaranteed to reach any old hollow), thumb tang, long, fine black rubber handles, fancy etching, honed and strapped. Regular price, $1.00, sold to you for fifty cents."

Jes' think of it, Rastus, a genteel and effective side-arm for only half a dollar!

Further on is a life-sized picture of a proud Sir Chanticleer, and freely insterspersed through the pages of the *Beacon-Light*, are other fowls both great and small, temptingly isolated from the immediate vicinity of the haunts of man.

We note with surprise the absence of even casual mention of watermelons. This is an oversight that is hard to explain on any other assumption than that it is deliberate, and grew out of the seasonal appropriateness of roast 'possum over watermelon. If we are honored with further copies, we confidently expect to see a special watermelon edition next summer—*an amende honorable* to indulgent patrons. Success to the *Beacon-Light*! May it safely guide the footsteps of many over woodpiles and barnyards to the haven of the "hennery" and as securely and expeditiously direct them thither.

---

### ANNOUNCEMENT OF NEW BOOKS.

The following books are in press and will soon be issued by the publishers, J. B. Flint & Co., 104 Fulton street, New York: "Flint's Encyclopedia of Medicine and Surgery." Second (1898) edition, 1555 pages, revised with the assistance of fifty-six contributors and thoroughly in line with recent advances in medical science. Cloth $5, Leather or Half Morocco $6. "Hartley-Auvard System of Obstetrics." Third (1898) edition, 426 pages, 543 illustrations. Revised by Dr. John D. Hartley.

This work is essentially Auvard, and embodies the author's personal experience; the text is clearly pictured by hundreds of original drawings to be found in no other book. Cloth $4, Leather or Half Morocco $5. "Pozzi System of Gynecology." Third edition. Revised by Dr. John D. Hartley.

---

### THE AMERICAN MEDICAL ASSOCIATION.

*Section on Materia Medica and Therapeutics*: The following papers and discussions have been promised for the meeting at Denver, Col., June 7–10, 1898:

"Yellow Fever: Its Etiology and Treatment." Discussion by Surgeon-General George M. Sternberg, M.D., of Washington, D. C.; Prof. John Guiteras, M.D., of Philadelphia; Sollace Mitchell, M.D., of Jacksonville, Fla.; T. S. Scales, M.D., of Mobile, Ala.; G. B. Thornton, M.D., of Memphis, Tenn.; H. M. Bracken, M.D., of Minneapolis, Minn.; P. E. Archinard, M.D., of New Orleans, La.

"Aims of Modern Treatment of Tuberculosis." By Prof. Edwin Klebs, M. D., of Chicago. Discussion by Charles Denison, M.D., of Denver, Col.; C. H. Whitman, M.D. of Los Angeles, Cal.

"Serum Therapy of Tuberculosis." By Prof. S. O. L. Potter, M.D., of San Francisco, Cal. Discussion by Prof. James M. Anders, M.D., of Philadelphia.

"The Therapeutics of Pulmonary Phthisic." By Paul Paquin, M.D., of St. Louis, Mo.

"Tuberculin as a Diagnostic and Curative Agent, with Report of 250 Tubercular Cases Treated." By C. H. Whitman, M.D., of Los Angeles, Cal.

"The Practical Value of Artificial Serum in Medical Cases." By P. C. Remondino, M.D., of San Diego, Cal.

"The Use of Remedies in Diseases of the Heart and Blood-vessels." By T. Lauder Brunton, M.D., D.Sc., F.R.S., London, England.

"The Mescal Button." By Prof. D. W. Prentiss, M.D., of Washington, D. C.

"The Modern Intestinal Antiseptics and Astringents." By William Frankhauser, M.D., of New York.

"To What Extent is Typhoid Fever Favorably Modified in Its Course, Duration, Termination or Sequelæ by the Administration of Drugs." By Frank Woodbury, M.D., of Philadelphia, Pa.

"Strychnine." By J. N. Upshur, M.D., of Richmond, Va.

"Methods of Teaching Materia Medica and Therapeutics." By Prof. G. H. Rohe, M.D., of Baltimore.

"The Study of Materia Medica and Therapeutics." By H. M. Bracken, M.D., of Minneapolis, Minn.

"The Great Therapeutic Importance of a Rational Adaptation of Cathartic Remedies to the Physiological Functions of the Gastro-Intestinal System." By E. D. McDaniels, M.D., LL.D., of Mobile, Ala.

"Why the Pharmacopœial Preparations Should be Prescribed and Used by the Profession." By Leon L. Solomon, M.D., of Louisville, Ky.

"The Use of Electricity by the General Practioner." By Caleb Brown, M.D., of Sac City, Ia.

The following have also promised papers, subjects to be announced very soon, together with the day assigned for each discussion and paper:

Dr. J. E. Atkinson, of Baltimore, Md.; Dr. Henry Beates, of Philadelphia, Pa.; Dr. T. M. Balliet, of Philadelphia, Pa.; Dr. George F. Butler, of Chicago, Ill.; Dr. Dudley W. Buxton, of London, Eng., Dr. J. Solis-Cohen, of Philadelphia, Pa.; Dr. N. S. Davis, Jr., of Chicago, Ill.; Dr. P. J. Farnsworth, of Clinton, Ia.; Dr. J. E. Moses, of Kansas, City, Mo.; Prof. Joseph Remington, of Philadelphia, Pa.; Dr. L. E. Sayre, of Lawrence, Kan.; Dr. H. V. Sweringen, of Fort Wayne, Ind.; Dr. E. L. Stephens, of Fort Worth, Texas.

The chairman will be pleased to receive and place upon the program subjects for discussion and papers.

JOHN V. SHOEMAKER, M. D., Chairman,
1519 Walnut street, Philadelphia, Pa.

